# Protocol for a feasibility trial for improving breast feeding initiation and continuation: assets-based infant feeding help before and after birth (ABA)

**DOI:** 10.1136/bmjopen-2017-019142

**Published:** 2018-01-23

**Authors:** Kate Jolly, Jenny Ingram, Joanne Clarke, Debbie Johnson, Heather Trickey, Gill Thomson, Stephan U Dombrowski, Alice Sitch, Fiona Dykes, Max G Feltham, Kirsty Darwent, Christine MacArthur, Tracy Roberts, Pat Hoddinott

**Affiliations:** 1 Institute of Applied Health Research, University of Birmingham, Birmingham, UK; 2 Centre for Child and Adolescent Health, University of Bristol, Bristol, UK; 3 Department of Social Medicine, University of Cardiff, Cardiff, UK; 4 University of Central Lancashire, Preston, Lancashire, UK; 5 Maternal and Infant Nutrition and Nurture Unit, University of Central Lancashire, Preston, UK; 6 Department of Psychology, University of Stirling, Stirling, UK; 7 Birmingham Clinical Trials Unit, University of Birmingham, Birmingham, UK; 8 Nursing, Midwifery and Allied Health Professional Research Unit, University of Stirling, Stirling, UK; 9 Health Economics Unit, University of Birmingham, Birmingham, UK

**Keywords:** public health, breastfeeding, Assets-based

## Abstract

**Introduction:**

Breast feeding improves the health of mothers and infants; the UK has low rates, with marked socioeconomic inequalities. While trials of peer support services have been effective in some settings, UK trials have not improved breast feeding rates. Qualitative research suggests that many women are alienated by the focus on breast feeding. We propose a change from breast feeding-focused interactions to respecting a woman’s feeding choices, inclusion of behaviour change theory and an increased intensity of contacts in the 2 weeks after birth when many women cease to breast feed. This will take place alongside an assets-based approach that focuses on the positive capability of individuals, their social networks and communities.

We propose a feasibility study for a multicentre randomised controlled trial of the Assets feeding help Before and After birth (ABA) infant feeding service versus usual care.

**Methods and analysis:**

A two-arm, non-blinded randomised feasibility study will be conducted in two UK localities. Women expecting their first baby will be eligible, regardless of feeding intention. The ABA infant feeding intervention will apply a proactive, assets-based, woman-centred, non-judgemental approach, delivered antenatally and postnatally tailored through face-to-face contacts, telephone and SMS texts. Outcomes will test the feasibility of delivering the intervention with recommended intensity and duration to disadvantaged women; acceptability to women, feeding helpers and professionals; and feasibility of a future randomised controlled trial (RCT), detailing recruitment rates, willingness to be randomised, follow-up rates at 3 days, 8 weeks and 6 months, and level of outcome completion. Outcomes of the proposed full trial will also be collected. Mixed methods will include qualitative interviews with women/partners, feeding helpers and health service staff; feeding helper logs; and review of audio-recorded helper–women interactions to assess intervention fidelity.

**Ethics and dissemination:**

Study results will inform the design of a larger multicentre RCT. The National Research Ethics Service Committee approved the study protocol.

**Trial registration number:**

ISRCTN14760978; Pre-results.

**Strengths and limitations of this study**

- This study uses a two-centre randomised controlled trial design to determine the feasibility of a definitive trial.
- The intervention design draws on evidence from best practice to support women who want to breast feed, behavioural change theory and makes use of women’s personal social and community assets.
- A process evaluation will explore reach, fidelity of intervention delivery and the experience of women, feeding helpers and other key stakeholders.
- The success of the study will depend on ability to deliver the intervention with sufficient fidelity.
- A definitive trial would be necessary to evaluate the effectiveness of the intervention.

## Introduction

Breast feeding is associated with health benefits for both the infant and mother^1–9^; however, breast feeding duration in the UK is among the lowest worldwide, with relatively small improvement over the past two decades,^10^ particularly for exclusive breast feeding. There are considerable health inequalities with breast feeding initiation and duration rates lowest in teenagers, socioeconomically disadvantaged women, women with lower educational levels and white women.^10^ Many women cease breast feeding before they plan to, with 8 out of 10 women who stop breast feeding in the first 2 weeks reporting that they would have liked to have breast fed for longer.^10^

WHO recommends exclusive breast feeding for 6 months,^11^ yet fewer than 1% of UK infants receive breast milk only for this period.^10^ The steepest decline in breast feeding occurs early: 81% initiate breast feeding; 66% breast feed at 2 weeks. Exclusive breast feeding rates are even lower: 46% at 1 week and 23% at 6 weeks. Mothers express dissatisfaction with breast feeding care^12 13^ and 30% report feeding problems in the early weeks.^10^ Women who report they did not receive support for breast feeding difficulties in hospital, or at home, were more likely to discontinue breast feeding at this stage.^10^

### Effectiveness of peer support for breast feeding initiation and continuation

Breast feeding peer support has been widely advocated in the UK as a means of increasing breast feeding initiation and continuation rates in women from disadvantaged communities.^14 15^ Peer support has been defined as ‘support offered by women who have received appropriate training and have either themselves breastfed or have the same socioeconomic background, ethnicity or locality as the women they are supporting’.^16^ A systematic review of breast feeding initiation^17^ reported a significant increase in three trials that targeted the support at pregnant women who had decided to breast feed, but no difference in three trials offering peer support to all pregnant women. A systematic review of breast feeding continuation^16^ reported significant effects of peer support (all settings) on any and exclusive breast feeding rates particularly when given at higher intensity (≥5 contacts). Peer support interventions had a significantly greater effect on any and exclusive breast feeding in low-income or middle-income countries compared with high-income countries. However, no significant effect on any or exclusive breast feeding was observed in the three UK-based studies.

A 2017 Cochrane review of support for breast feeding mothers^18^ found nine trials of lay support compared with usual care and reported a lower risk of stopping breast feeding before last study follow-up compared with usual care, but the interventions and settings were heterogeneous. In the previous Cochrane review,^19^ five trials offered peer support requiring women to initiate contact; none of these increased breast feeding rates, suggesting that to be effective, peer support should be proactively offered. Peer supporters in a Canadian trial proactively telephoned women using an unstructured format,^20^ and increased breast feeding rates at 4 weeks compared with controls. Preliminary research suggests that proactive early telephone support might suit a UK context. A pilot trial showed intensive early proactive telephone support for women who initiated breast feeding delivered by a postnatal ward feeding team with personal breast feeding experience increased any breast feeding by 22% at 6–8 weeks compared with the non-proactive opportunity to access telephone support from the team.^21^

A recent UK study of barriers to effective lay feeding help recommended that to gain wider acceptability, interventions should be (1) women centred (rather than breast feeding centred), both enabling breast feeding and helping with formula milk feeding; (2) have most focus on the early weeks (when breast feeding is being established and women often stop breast feeding before they planned); and (3) offered proactively.^22^ This supports qualitative insights that recommend person-centred^23^ flexible approaches.^24^ How breast feeding interventions are delivered and the intervention–context fit are important determinants of outcomes.^25^ Early support may be an important feature of effective breast feeding support.^26 27^

### Information needs and risks in mothers who feed their babies formula milk

Evidence shows that for interventions to be acceptable, it is important to address issues related to mixed and formula feeding.^22 28^ The latest (2010) Infant Feeding Survey showed that 54% of babies had received formula milk by 1 week and the vast majority had received at least some formula milk in their first year.^10^ It highlighted that half of mothers who prepared powdered infant formula did not follow all three key recommendations intended to reduce risk of infection and overconcentration of feeds. Other authors have also highlighted a high frequency of errors in formula feed preparation.^29 30^ Evidence indicates that an intervention to increase breast feeding which fails to address mothers’ needs in relation to formula feeding, particularly in a culture where mixed feeding is common, risks alienating potential beneficiaries, limiting intervention reach and retention, and decreases the likelihood of achieving breast feeding-related outcomes.^31^ In addition, safe formula feeding practices should reduce infections in formula-fed babies.

### Assets-based approaches in public health

An assets-based approach focuses on the positive capability of individuals and communities, rather than solely on their needs, deficits and problems. It is essentially about recognising and making the most of people’s strengths, to ‘redress the balance between meeting needs and nurturing the strengths and resources of people and communities’,^32^ with a corresponding shift in focus from determinants of illness to determinants of health and well-being. Although assets can include material resources,^32 33^ in public health, more typically, the primary focus is on valuing individual and collective psychosocial attributes. These include self-esteem, confidence, optimism, knowledge and skills, as well as features of social capital such as social networks and reciprocity.^34–36^

In the context of breast feeding and well-being, assets are likely to include intrinsic personal resources, particularly self-efficacy in relation to feeding, motivation and drive to maintain feeding, and the willingness to ask for and accept help. Extrinsic resources having an influence include availability of social support from partner,^37 38^ family and friends; wider social networks of other women who have breast fed; and community assets such as breast feeding groups or baby cafes, children’s centres and mother/baby groups. Local peer supporters are also community assets for breast feeding.

### Rationale for the Assets feeding help Before and After birth (ABA) study

The ABA intervention is a woman-centred feeding helper approach built on systematic review evidence,^16 17 19 23^ behaviour change theory^39^ and extensive qualitative research.^23 24 28 40^ It takes an assets-based approach, enabling support to be tailored to a woman’s individually available assets for breast feeding.

The intervention aims to establish a strong supportive helper–woman relationship with continuity of care from pregnancy until after birth, respect a woman’s choices, and be non-judgemental and offer discussion of breast feeding and formula feeding issues. This new broader ‘feeding’ approach is compliant with Unicef UK guidance,^41^ but without alienating women considering mixed or formula feeding by using the term ‘breast feeding’ in promotional material and support provided.^28 42 43^

### Aims and objectives

The overall aim is to assess the feasibility of delivering a new ABA feeding helper intervention within a randomised controlled trial. Detailed objectives are listed in box.

Box **Detailed study objectives**

1. To adapt existing peer support services to provide a new infant feeding helper intervention, underpinned by theory and evidence, with service user and provider input.
2. To undertake a feasibility randomised controlled trial (RCT) of the new feeding helper role compared with usual care (control group) for women living in areas of low breast feeding prevalence.
3. To determine levels of uptake and engagement with the intervention; to describe socioeconomic/demographic profiles to ascertain reach and explore health inequalities.
4. To describe care received by the reactive ‘usual care group’ in relation to feeding method.
5. To assess fidelity of intervention delivery and any contamination, and to explore feedback from feeding helpers to improve fidelity if required.
6. To assess whether women are willing to be recruited and randomised, whether the expected recruitment rate for a subsequent full-scale effectiveness RCT is feasible and to identify successful recruitment strategies.
7. To explore mothers’ and feeding helpers’ perceptions of the intervention, trial participation and processes.
8. To explore acceptability and fidelity of the intervention when delivered by paid and volunteer feeding helpers.
9. To assess acceptability and integration of the intervention to other providers of maternity, postnatal care and social care.
10. To explore the relative value of the individual feeding support versus the community integration elements to inform the design of a future trial.
11. To provide estimates of the variability in the primary outcome to enable sample size calculation for a definitive trial.
12. To measure the features of the feeding helper provision and service use, which would underpin the cost-effectiveness of the intervention and determine the feasibility of data collection.
13. To test components of the proposed RCT to determine feasibility of the protocol.

## Methods and analysis

The design is informed by the (Medical Research Council) MRC Complex interventions and RE-AIM frameworks.^44 45^ The SPIRIT^46^ (Standard Protocol Items for Randomised Interventional Trials) checklist was used to inform the content of this protocol.

The Public and Researchers Involvement in Maternity and Early Pregnancy (PRIME) group were involved in developing this protocol. Lay co-applicants BM and JK-C (previous chair of PRIME Group) are members of the trial management group and will provide a service user perspective to the study. They will contribute to intervention development and participant facing materials. In addition, women identified through children’s centres are being consulted at key decision points and will be involved in co-production of the intervention text messages.

### Study design

ABA is an individually randomised controlled trial (RCT) in two UK sites with the intervention delivered by ABA feeding helpers (paid and volunteer).

**Figure 1:**
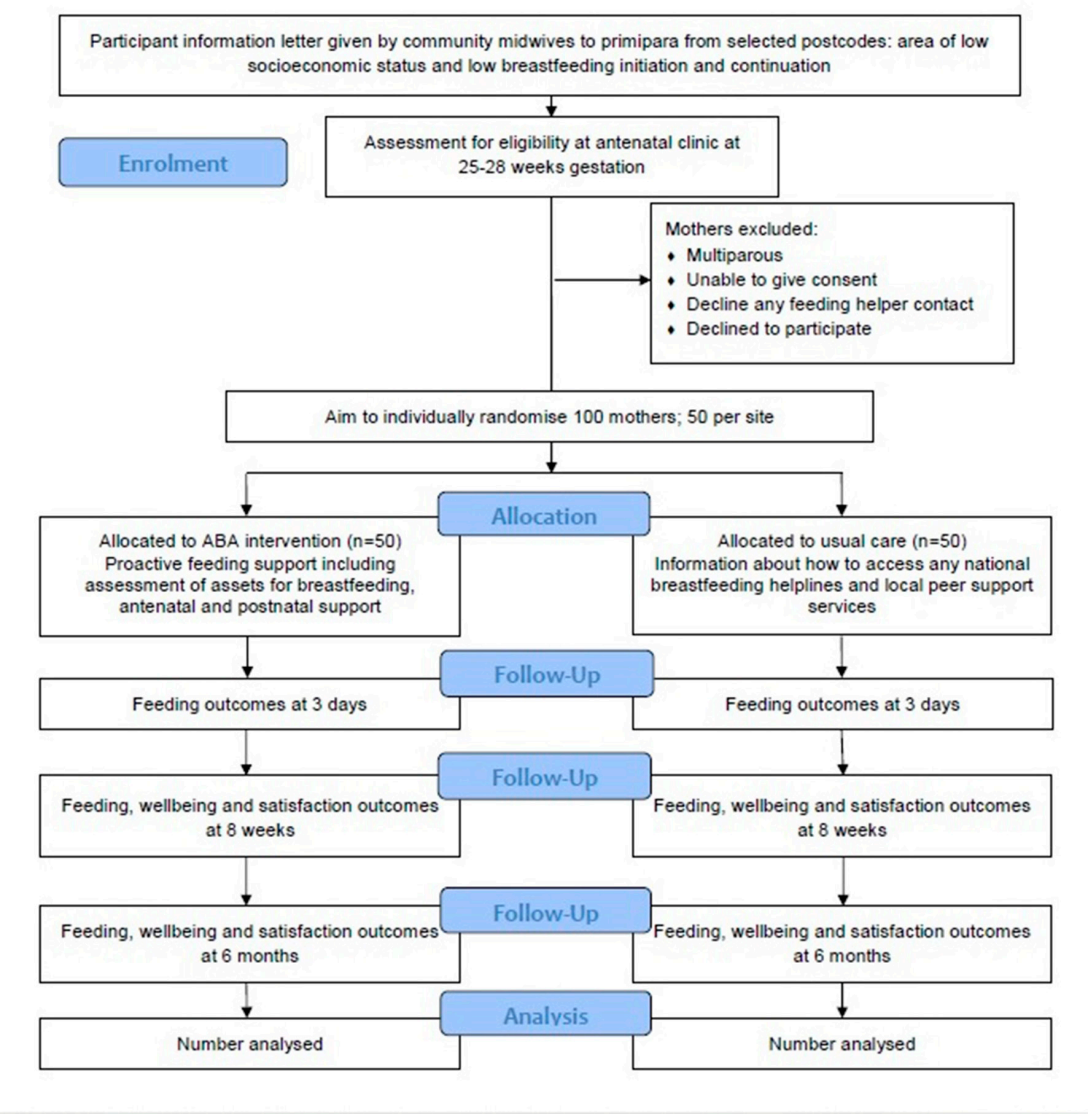
Study flow diagram.

A mixed-methods process evaluation will take place alongside the trial to measure reach; intervention fidelity; acceptability to mothers, helpers and professionals; experiences of feeding helpers and integration of the service with midwives, health visitors and other social care providers. Qualitative interviews with professionals, feeding helpers and mothers will be conducted to understand the relative role of potential intervention mechanisms operating at individual, service and community levels. Key stages of the trial are detailed in figure 1.

### Study population, setting and recruitment plan

The trial will take place in two geographical areas with existing peer support programmes, but where the service is currently offered on a reactive basis, that is, self-referral or midwife referral. Sites were selected with low breast feeding initiation/continuation rates in areas of relatively high socioeconomic disadvantage. One site has paid peer supporters (site A); one uses volunteers (site B), reflecting the diversity of UK peer support services.

Women are eligible for inclusion, regardless of feeding intention, if they are aged 16 years or older, pregnant with their first child and residing in the study locality.

Women are handed information about the study by their community midwife at an antenatal clinic and then approached at an antenatal appointment by a research fellow who will take informed consent from women willing to participate.

Randomisation will be undertaken differently at the two sites to enable a controlled flow of participants to the volunteer peer supporters at site B.

At site A, a block randomisation list has been developed by an independent statistician, stratified by age group (<25/≥25 years) and held in a secure database unavailable to those who enrol participants or assign interventions. Once a woman consents to the trial and the baseline case report form completed, the research fellow will telephone the randomisation system. The research fellow will inform the woman of her allocation at the clinic or, if not available, by letter.

At site B, a Microsoft Access database has been developed by a clinical trials unit and used to randomise blocks of women from each site subarea. Each block will be randomised simultaneously. If an odd number of cases are recruited, the allocation will be biased towards the intervention. This randomisation procedure is needed to ensure that the numbers allocated to receive the intervention matches the number of volunteers available for intervention delivery and their capacity to deliver the intervention. Randomisation will be undertaken by an independent researcher.

### Planned interventions

#### Usual care group

The comparator group will receive usual care including midwife and health visiting support. The feeding support available and accessed by women will be described, including local services such as peer supporters and any breast feeding support groups, and national breast feeding helplines, but these will only be available reactively (the woman initiates the contact or a midwife does so on the woman’s behalf).

#### Intervention group

##### Intervention rationale and design

**Figure 2:**
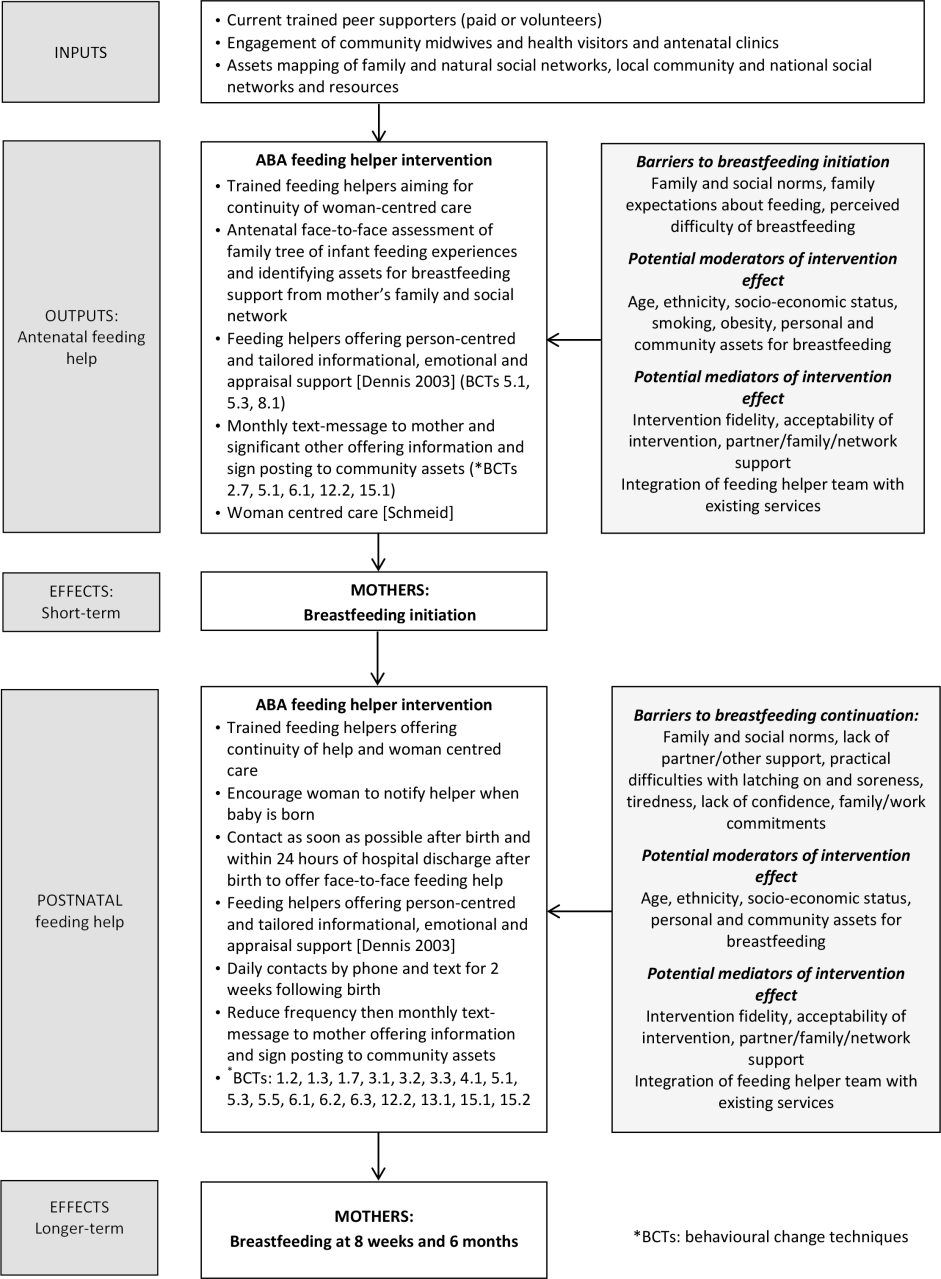
Logic model for Assets feeding help Before and After birth (ABA) study.

The intervention is proactive support, underpinned by an assets-based approach. It provides person-centred care^23^ and best evidence in relation to settings, frequency, duration and manner of providing support from an ‘ABA infant feeding helper’. A logic model of the intervention is detailed in figure 2. The intervention commences antenatally (approximately 30 weeks) and can continue until 5 months after birth.

Prior to the start of the feasibility trial, national information, helplines, social media resources and local feeding health and community ‘assets’ such as antenatal and postnatal groups and baby cafés were mapped as a choice menu in a leaflet which had input from our PPI group.

During the antenatal face-to-face meeting between the woman and ABA feeding helper, personal assets in terms of family, friends and social networks will be identified. Women will be encouraged to draw on these assets to enhance their capacity to achieve their feeding goals.

To inform the intervention, we used information from systematic reviews, surveys, qualitative studies and PPI discussion to identify barriers to breast feeding initiation and continuation. The behaviour change wheel framework (behaviours analysed in terms of the capability, opportunity and motivation; COM-B) in conjunction with the theoretical domains framework was used to identify a range of behaviour change functions and techniques from the behaviour change taxonomy.^39 47^ Potential techniques were then analysed using the APEASE criteria (affordability, practicability, effectiveness, acceptability, equity)^48^ to identify intervention components, which were simple, cheap, practical and acceptable. A review of multicomponent incentive interventions to support breast feeding mapped behaviour change techniques and found that social support dominated^49^ and is a key concept underpinning peer support.^50^ The final behaviour change techniques selected are detailed in table 1 with further details of the process in online supplementary appendix 1.

10.1136/bmjopen-2017-019142.supp1 Supplementary file 1

**Table 1:** Behaviour change techniques (BCTs)

BCT no	Label	Definition	Examples
1	Goals and planning		
1.2	Problem solving	Analyse or prompt the person to analyse factors influencing the behaviour and generate or select strategies that include overcoming barriers and/or increasing facilitators.	Prompt the woman to consider what may encourage or prevent them from successful breast feeding. Help the woman to identify strategies, solutions and support they can access to help overcome any difficulties.
1.3	Goal setting (outcome)	Set or agree on a goal defined in terms of a positive outcome of wanted behaviour.	To discuss the woman’s (postnatal only) goals for breast feeding.
1.7	Review outcome goal(s)	Review outcome goal(s) jointly with the person and consider modifying goal(s) in light of achievement. This may lead to re-setting the same goal, a small change in that goal, or setting a new goal instead of, or in addition to, the first.	To have ongoing discussions about the woman’s breast feeding achievements and to provide support for alternatives (ie, mixed feeding, breast feeding cessation) as appropriate.
2	Feedback and monitoring		
2.7	Feedback on outcome(s) of behaviour	Monitor and provide feedback on the outcome of performance of the behaviour.	Inform the woman about ongoing health benefits of breast feeding at different stages.
3	Social support		
3.1	Social support (unspecified)	Advise on, arrange or provide social support (eg, *from friends, relatives, colleagues, ‘buddies’ or staff*) or non-contingent praise or reward for performance of the behaviour. It includes encouragement and counselling, but only when it is directed at the *behaviour*.	Suggest that the woman calls a ‘buddy’ if they feel they are struggling with feeding or need some support. Provide positive feedback on woman’s progress with breast feeding. Arrange for a family member or friend to encourage continuation with breast feeding.
3.2	Social support (practical)	Advise on, arrange or provide *practical* help (eg, *from friends, relatives, colleagues, ‘buddies’ or staff*) for performance of the behaviour.	Suggest the woman call an infant feeding helper, health professional, helpline or ‘buddy’ if they feel they are struggling with feeding or need some support. Ask the partner/family members of the woman to bring her the baby when it is ready to feed, bring a drink for the mother. Ask the partner/family members to help with other activities in the home while the mother is feeding the baby (meal preparation, washing). Encourage the woman to access a breast feeding support group or to call a helpline during times when other people are not available to help.
3.3	Social support (emotional)	Advise on, arrange or provide *emotional* social support (eg, *from friends, relatives, colleagues, ‘buddies’ or staff*) for performance of the behaviour.	Ask the woman to take a friend to the breast feeding group or ask the feeding helper to meet her there.
4.	Shaping knowledge		
4.1	Instruction on how to perform a behaviour	Advise or agree on how to perform the behaviour (includes ‘*Skills training*’).	Provide information (visual images) and model demonstrations to show the woman how to position her baby to facilitate good latching on. Show a woman how to prepare a bottle of formula correctly.
5.	Natural consequences		
5.1	Information about health consequences	Provide information (eg, written, verbal, visual) about health consequences of performing the behaviour.	Explain the health benefits of breast feeding to both the woman and baby.
6.	Comparison of behaviour		
6.1	Demonstration of the behaviour	Provide an observable sample of the performance of the behaviour, directly in person or indirectly for example, via film, pictures, for the person to aspire to or imitate.	Demonstrate breast feeding in film clip or via the use of aids (eg, breast feeding doll). Pictures of ‘good’ positioning and attachment to be shared with women. Encourage attendance at breast feeding group to observe other women breast feeding.
8	Repetition and substitution		
8.1	Behavioural practice/rehearsal	Prompt practice or rehearsal of the performance of the behaviour one or more times in a context or at a time when the performance may not be necessary in order to increase habit or skill.	Show and ask women to practice behaviours (ie, hand expressing or breast feeding) using aids such as a breast feeding doll or knitted breast.
12.	Antecedents		
12.2	Restructuring the social environment	Change or advise to change the *social* environment in order to facilitate performance of the wanted behaviour.	Encourage the woman to attend social gatherings where other mothers are breast feeding.
13.	Identity		
13.1	Identification of self as role model	Inform that one’s own behaviour may be an example to others.	Inform the woman that if they breast feed, they will be a role model within their community and to their child who will be influenced by their feeding choice.
15.	Self-belief		
15.1	Verbal persuasion about capability	Tell the person that they can successfully perform the wanted behaviour, arguing against self-doubts and asserting that they can and will succeed.	Inform the woman that they can successfully breast feed despite initial difficulties. Encourage women to talk to friends/family members as well other mothers at breast feeding groups to hear stories of how others have managed to breast feed successfully.
15.2	Mental rehearsal of successful performance	Advise to practice imagining performing the behaviour successfully in relevant contexts.	Ask and encourage women to imagine breast feeding in public locations and plan how this can be undertaken discretely.

The intervention will commence antenatally. The ABA feeding helper will telephone women at about 30 weeks and offer a face-to-face discussion at home or location of their choice (eg, children’s centre or cafe) to discuss infant feeding and explore their assets for breast feeding. Local policies for home visiting by the feeding helpers will be taken into consideration. A narrative storytelling approach will be used to produce a family tree diagram (genogram) of infant feeding experiences, widening to the natural social network^51^ to enable women to reflect on future feeding relationships.^52^ This will allow breast feeding to be introduced in a woman-centred rather than promotional way. Partners/family members will be encouraged to be present so their support role can be emphasised and encouraged.

Further follow-up will be by monthly texts during pregnancy; the key aim is to establish continuity of care and strong rapport between woman and ABA feeding helper to enable effective engagement immediately after birth.

ABA feeding helpers will encourage women to let them know as soon as convenient after the birth by swapping mobile phone numbers, encouraging them to be on the list of people notified after the birth and by the use of a fridge magnet with contact details. The objective is for the feeding helper to telephone within 24 hours of the woman going home and offer an early face-to-face meeting.

Subsequent support will be by brief daily telephone call/texts until the baby is 2 weeks^21^ then reducing in frequency up to 8 weeks based on maternal preference, with final texts at 3, 4 and 5 months. The ABA feeding helpers will be able to choose from a library of texts co-produced with mothers and informed by relevant literature^13 23 24 28 40 42 43^ with embedded behavioural change techniques. Home visits/meetings in community venues can also be organised as required. Women can request that texts and calls stop at any point.

In site A, the intervention will be delivered by paid peer supporters employed by a social enterprise organisation. This service currently provides postnatal breast feeding support by phone and home visits to women referred from professionals, peer supporters in postnatal wards and self-referrals.

In site B, volunteer breast feeding peer supporters managed by a national charity will deliver the intervention. These peer supporters currently volunteer in children’s centres by attending breast feeding groups and provide no proactive antenatal support.

#### Training

Six hours of training will be provided to peer supporters on intervention delivery, covering intervention goals, how the role of the ABA feeding helper differs from breast feeding peer support, active listening, key messages underlying the intervention and myths/truths about formula feeding. Skills will be developed through observation of modelled interactions, practising using the genogram and role play of scenarios, including how to discuss local services and other available support. Training was developed and will be delivered by HT and KD.

### Outcomes

#### Feasibility outcomes

Our main feasibility outcomes are to assess the feasibility of delivering the intervention and the research methods by (1) reach of recruitment of women to reflect required sociodemographic profile; (2) ability to recruit, train and engage current peer supporters to the new ABA feeding helper role; (3) ability to deliver planned number of contacts at a time and location convenient for participants; (4) acceptability to women; (5) fidelity of delivery and whether woman-centred care was provided; (6) unintended consequences of the intervention; (7) the feasibility of a future definitive trial assessed by recruitment rates, willingness to be randomised, follow-up rates at 3 days, 8 weeks and 6 months and level of completion of assessments by text^53^ (see criteria for progression to main trial) and (8) potential cases of intervention contamination in the control group: at 8 weeks of follow-up, all women will be asked about the use of national breast feeding helplines, any breast feeding support, whether there was a home visit or one-to-one meeting at a children’s centre and number of contacts by the feeding helpers. They will be asked in interviews whether they met other women taking part in the study and whether they had discussed the study.

The feasibility RCT will assess whether the whole trial can be run as planned and will include outcome measures that a definitive trial would collect. Particular attention will be paid to levels of missing data and contamination. Criteria for progression to a main trial are below.

#### Outcomes measures for a definitive trial included in the feasibility trial

Primary outcome of future definitive trial: any breast feeding at 8 weeks.

Secondary outcomes: breast feeding initiation (at 2–3 days defined in accordance with the UK Infant Feeding Survey^10^ as putting the baby to the breast, even if on one occasion only and includes giving expressed breast milk); exclusive breast feeding at 6–8 weeks and any/exclusive breast feeding at 6 months, if ceased breast feeding, duration of any and exclusive breast feeding (exclusive breast feeding defined in accordance with WHO definition of infants who received only breast milk during the previous 24 hours^54^). Maternal well-being (Warwick-Edinburgh Mental Well-being Scale)^55^ and maternal satisfaction with feeding experience and support provided at 8 weeks and 6 months, using a single-item question used in a previous trial^21^ and co-produced with PPI.

Outcomes relevant to future economic evaluation: self-reported use of health and feeding support services will be asked in the 8-week questionnaire. Overall feeding support activity during the intervention period will be obtained from logs (feeding helpers and local peer supporters). At 8 weeks and 6 months, use of childcare will be collected by questionnaire. Qualitative interviews with women will explore whether there are costs to family or social networks in supporting a woman in her breast feeding as these would need to be considered by a societal perspective in a future economic evaluation.

### Process evaluation

The process evaluation will address programme reach, the fidelity of delivery by feeding helpers, use of local and personal assets for feeding support, mothers’ views of the ABA feeding helper intervention, views of feeding helpers and of other providers of maternity services, and the presence of social desirability bias. Details are shown in table 2.

**Table 2:** Process evaluation

Process measure	Assessed by:
Programme reach	Uptake (from recruitment rate); Randomisation; Retention; Characteristics of women recruited (age, ethnicity, living arrangements, index of multiple deprivation, education and employment) Follow-up data at 8 weeks and 6 months.
Fidelity of delivery by feeding helpers	Analysis of the content of recorded face-to-face and text interactions between feeding helpers and mothers; Activity logs kept by feeding helpers; Qualitative interviews with feeding helpers and women in the intervention group to triangulate data.
Use of local and personal assets for feeding support	Analysis of the content of recorded face-to-face and telephone discussions between feeding helpers and mothers; Qualitative interviews with both feeding helpers and women.
Mothers’ views of the ABA feeding helper intervention and acceptability	Qualitative interviews with the mothers (approximately 20 intervention and 10 usual care).
Views of feeding helpers in relation to training, acceptability and satisfaction	Qualitative interviews (all feeding helpers will be invited to be interviewed).
Views of other providers of maternity services in relation to integration of the intervention with other support offered to women	Through telephone interviews with a range of professionals/service providers (n=12).
Presence of social desirability bias:	Feeding helper logs; Text messages; Recorded interactions; Interviews with mothers and feeding helpers; Routine feeding status data.

ABA, Assets-based infant feeding help Before and After birth.

### Assessment and follow-up

At baseline (approximately 25–28 weeks of gestation), we will collect brief demographic characteristics. To collect initiation data (at 2–3 days), we will pilot the use of text messages, telephone calls and emails.^53^ Women will be sent a brief questionnaire at 8 weeks and 6 months postnatally to collect method of infant feeding and additional data. To try to maximise follow-up rates, data will be triangulated with routinely collected data on initiation and feeding status at 6–8 weeks and women will be offered a £25 ‘thank you’ voucher if they complete all the follow-ups.

### Assessment of harms

Women who have suffered fetal loss, perinatal death, whose baby is very ill or women who are seriously ill after a difficult birth are likely to be distressed if they are approached by a feeding helper or the ABA research team. We will record and investigate any cases where women have been inappropriately contacted. Qualitative interviews will also explore unintended consequences.

### Data collection and management

Women will be asked to give consent to participate in the study and for their personal data to be transferred from site to the central study office. Participant names will not included on follow-up questionnaires. All identifiable data transferred will be subject to the appropriate informational governance protocol. Site files containing questionnaires and relevant documents as stipulated by local research and development and governance guidelines will be maintained throughout the study and securely stored in locked cabinets. Participant contact details will be transferred from the contact details form to a specifically designed study database, which is password protected and held on a secure server. Non-identifiable quantitative data will be transferred from the questionnaires to a specifically designed database. On study completion, all records created by following trial procedures and all documents listed in guidance relating to the conduct of the trial will be retained and archived for a period of 10 years from the end of the study, in accordance with the University of Birmingham Code of Practice for research.

### Sample size

Sample size was chosen to enable estimation of feasibility outcomes with reasonable precision. We will be able to estimate recruitment, follow-up and questionnaire completion rates to within ±15% with 95% CI, based on a worst-case estimate of 50% for each outcome (target is 75%, 75% and 70%, respectively).

To inform the sample size calculation for a future definitive trial, we will calculate percentages of women initiating breast feeding and breast feeding at 8 weeks in the intervention and control groups; 95% CIs will be provided for estimates obtained.^56^

We aim to recruit at least 50 women at each site to achieve an overall sample size of 100, with half randomised to our intervention group. If in one group the percentage of women breast feeding at 6–8 weeks was 44%, a 95% CI for this estimate would range from 30.0% to 58.7%. For the percentage of women initiating breast feeding, an estimate may be 60% with a corresponding 95% CI of 45.2% to 73.6%. We wish to recruit sufficient teenagers, women of low socioeconomic status and women with a low social network experience of breast feeding, to ensure that their experience of the intervention is investigated.

### Statistical analysis

The statistician will be blinded to study allocation. We will report recruitment and follow-up rates, with 95% CIs, as a measure of feasibility of the trial.

The number and mode of ABA feeding helper and peer support contacts for both intervention and control groups will assess intervention implementation and contamination levels in the control group.

Although the trial is not powered to detect a difference between intervention and usual care groups, we will calculate the percentage of women breast feeding and exclusively breast feeding at 8 weeks for those allocated to each group; 95% CIs will be provided for estimates obtained. We will also evaluate dropout and data completeness for the feasibility study. This will inform the sample size calculation and which outcomes can feasibly be measured in a future definitive trial. Participant characteristics will be reported by randomisation group and simple summaries provided for each recorded outcome measure.

### Qualitative research methods, data management and analysis

Qualitative interviews with women will take place postnatally in the woman’s home, at a convenient location, or via telephone or Skype. We aim to include approximately 15 women at each site to capture a diversity of experience and may also conduct follow-up interviews with information-rich participants. Women can have someone of their choice present during the interview, as this can increase willingness to participate among women from socioeconomically disadvantaged groups. Sampling will be purposive, aiming for a diverse sample including teenagers, women in socioeconomically disadvantaged areas and women who have experienced different feeding journeys (ie, primarily formula feeding, breast feeding, mixed feeding). We will include women whose contact with the feeding helper has been high or low. Women in the usual care group whose 8-week questionnaire suggests that intervention contamination might have occurred will be selected for interview.

Interviews with intervention women will explore their experience and acceptability of ABA, and how the intervention interacts with other support sources particularly in relation to community assets (eg, breast feeding support groups, mother and baby groups). Interviews with ‘usual care’ women will explore their experiences of ‘usual care’ feeding support, acceptability of randomisation to the control group and any instances of contamination.

Qualitative interviews or focus groups with all feeding helpers will explore intervention acceptability and satisfaction in relation to the training they received, intervention delivery, barriers and facilitators to take-up, and intervention fidelity. Any unmet training/supervision needs will be identified. Causes of intervention contamination as perceived by ABA feeding helpers will be gathered.

Twelve qualitative telephone interviews with maternity care providers, including midwives, health visitors and children’s centre staff, will explore referral or delivery issues and the experience of integration of the feeding helpers into the wider early years services. We will explore issues of contamination (eg, whether they/colleagues have adopted the ABA approach or materials with women in the usual care group) and whether the intervention had any impact on how ‘usual care’ was provided. Professionals’ perceptions of the impact of the community assets-based element will also be sought.

Semistructured interview schedules based on the research literature, discussion within the team and input from PPI will be informed by our logic model and by the stages of breast feeding peer support intervention design model constructed from a realist review of experimental studies of peer support.^31^ All interviews will be recorded and transcribed verbatim. Reflective notes will be made following each interview.

We will use the framework approach to data management and analysis for the interviews and focus groups.^57^ A sample of transcripts will be read and re-read by researchers independently to develop an initial coding matrix of themes and categories. This will be discussed, refined and agreed before the remaining transcripts are analysed using the agreed coding framework. NVivo coding software will be used. Researchers will agree the coding framework and work collaboratively on the analysis. All data will be anonymised and any potentially identifying features removed.

### Economic component

The exploration of feasibility of appropriate data collection for the purpose of a future economic evaluation, in this trial, is restricted to exploring the achievability of collecting all health-service-related resource use associated with providing the intervention. This will show how possible it will be to estimate all health service costs associated with the intervention appropriately (eg, training ABA feeding helpers, telephone calls, text messaging service, one-to-one meetings with mother, staff time to respond to requests via text message and payments to peer supporters). Any future economic evaluation will be presented in terms of the additional cost per additional case of breast feeding for the intervention compared with usual practice. Future economic evaluation may consider the appropriateness of linking the intermediate outcome of an increase in the uptake of breast feeding to the longer-term health benefits using a model-based economic evaluation.

### Criteria for progression to a main trial

For the phase III trial to be considered, the following criteria need to be met: (1) process evaluation suggests that the intervention is acceptable to a majority of mothers, their partners, feeding helpers and local services; (2) recruitment of at least 75 women in 5 months; (3) able to recruit women of low socioeconomic status, teenagers and women from ethnic minority groups; (4) intervention implemented with fidelity in 75% of mothers (defined as contacts made in both the antenatal and postnatal period); (5) 75% receiving the assets-based antenatal face-to-face contact; and (6) >70% follow-up at 8 weeks and 6 months with ability to obtain additional missing data from routine sources.

The level of contamination of the usual care arm will inform whether an individually randomised trial would be feasible or whether a cluster RCT would be necessary. A cluster RCT would also be considered necessary if qualitative interviews confirm that significant contamination occurs or integration at a community level is a key mechanism of action, making individual randomisation impossible.

## Ethics and dissemination

### Monitoring and oversight

Aspects of the trial will form part of a portfolio of studies hosted and managed by the Women’s Health team at the Birmingham Clinical Trials Unit, University of Birmingham. The University of Birmingham holds the relevant insurance for this study and is the nominated sponsor for this study.

A trial steering committee (TSC) has been convened to provide overall supervision of the trial and ensure it is in accordance with the principles of good clinical practice and relevant regulations. The TSC agreed the trial protocol and will agree any protocol amendments. The TSC also provide advice to the investigators on all aspects of the trial including aspects of safety and monitoring of serious adverse events. The TSC is chaired by Professor Angela Harden (University of East London). Dr Amy Brown (University of Swansea), Dr Gulnaz Iqbal (University of Warwick) and Mrs Rebecca Jennings (lay representative) are members.

The intervention will adhere to policies and quality standards of the participating local authorities.

## Dissemination

A lay summary of the study is available on the National Institute for Health Research website. Final results of this feasibility study will be publicly available through open-access publication in a peer-reviewed journal and presented at relevant conferences and research meetings. The PPI groups will contribute to the dissemination plan.

## Supplementary Material

Reviewer comments
