# Supplementary material for: Protocol for a feasibility trial for improving breast feeding initiation and continuation: assets-based infant feeding help before and after birth (ABA)
Source: BMJ Open. 2018 Jan 23;8(1):e019142. doi: 10.1136/bmjopen-2017-019142 (PMC5786123; doi:10.1136/bmjopen-2017-019142)
Supplement: Supplementary file 1 [file bmjopen-2017-019142supp001.pdf]

Appendix 1 Intervention components: rationale for inclusion

Behaviour change item	COM-B component	Behaviour change techniques	Mode of delivery	Intervention function
Discuss benefits of breastfeeding	Motivation	Information about health consequences (individual)<br>Goal setting (outcome)	Face-to-face	Education,
Video-clip about breastfeeding	Motivation	Information about health consequences (general)<br>Mental rehearsal of behaviour<br>Instruction on how to perform the behaviour	Internet link from phone	Education,<br>Persuasion<br>Enablement
Breastfeeding support groups/social groups	Social opportunity<br>Capability<br>Motivation	Social support<br>Rehearsal (mental or actual) of behaviour<br>Verbal persuasion about capability<br>Demonstration of behaviour<br>Instruction on how to perform the behaviour<br>Restructuring the social environment	Face-to-face<br>Social media	Education,<br>Persuasion<br>Enablement
Written and web-site materials about feeding	Motivation	Information about health consequences<br>Instruction on how to perform the behaviour	Leaflet<br>Study web-site	Education,<br>Persuasion<br>Enablement
Identification of social network, social comparison, other facilitators and barriers to breastfeeding/support to overcome them	Capability<br>Social opportunity	Social support<br>Problem solving	Face-to-face	Enablement
Further telephone contact	Capability<br>Motivation	Social support<br>Feedback on outcome(s) of behaviour<br>Verbal persuasion about capability<br>Problem solving<br>Review outcome goal(s)<br>Identification of self as role model	Telephone	Enablement<br>Persuasion<br>Education
