# Supplementary material for: Protocol for a feasibility trial for improving breast feeding initiation and continuation: assets-based infant feeding help before and after birth (ABA)
Source: BMJ Open. 2018 Jan 23;8(1):e019142. doi: 10.1136/bmjopen-2017-019142 (PMC5786123; doi:10.1136/bmjopen-2017-019142)
Supplement: Reviewer comments [file bmjopen-2017-019142.reviewer_comments.pdf]

PEER REVIEW HISTORY

BMJ Open publishes all reviews undertaken for accepted manuscripts. Reviewers are asked to complete a checklist review form (**http://bmjopen.bmj.com/site/about/resources/checklist.pdf**) and are provided with free text boxes to elaborate on their assessment. These free text comments are reproduced below.

ARTICLE DETAILS

**TITLE (PROVISIONAL)**	Protocol for a feasibility trial for improving breastfeeding initiation and continuation: Assets-based infant feeding help Before and After birth (ABA)
**AUTHORS**	Jolly, Kate; Ingram, Jenny; Clarke, Joanne; Johnson, Debbie; Trickey, Heather; Thomson, Gillian; Dombrowski, Stephan; Sitch, Alice; Dykes, Fiona; Feltham, Max; darwent, Kirsty; MacArthur, Christine; Roberts, Tracy; Hoddinott, Pat

This article was not externally reviewed at BMJ Open. Protocols that have been independently assessed prior to submission to BMJ Open are usually fast-tracked to publication on the grounds that further substantial changes will not be possible. This independent assessment will usually be external, independent review for both a substantial grant award from a non-commercial or government funder and ethics approval.
